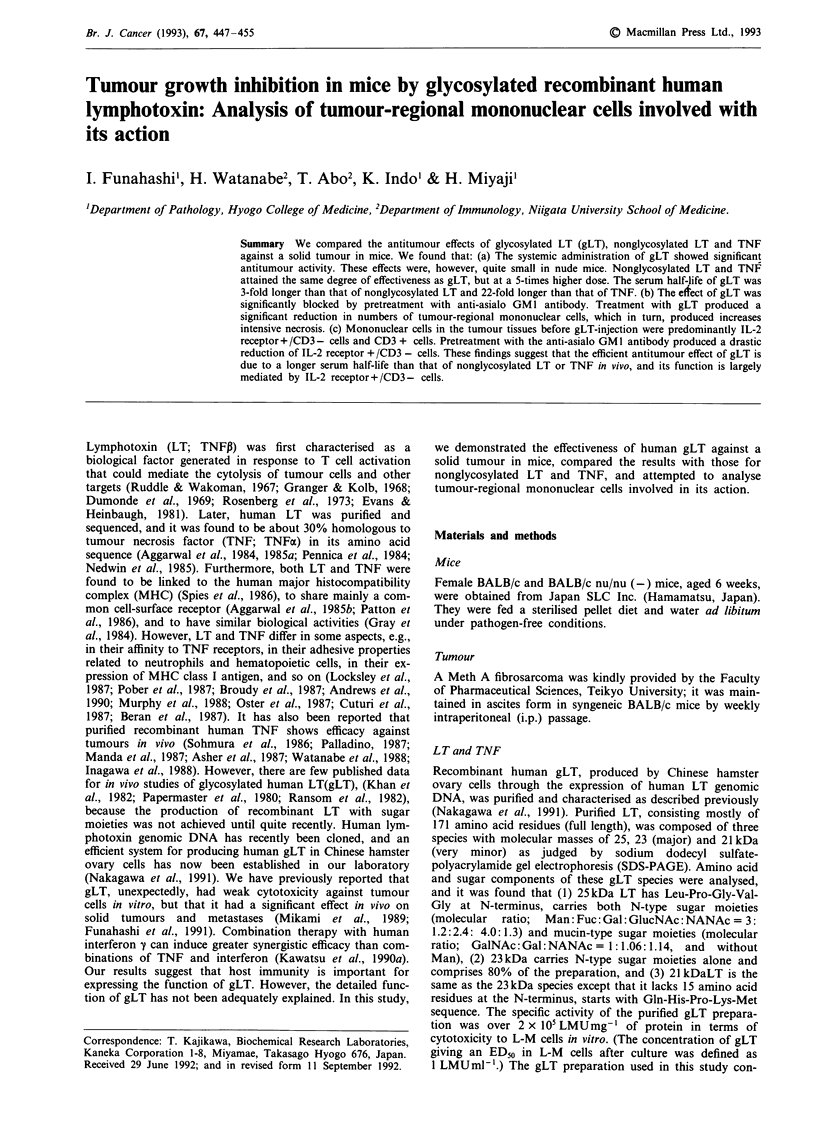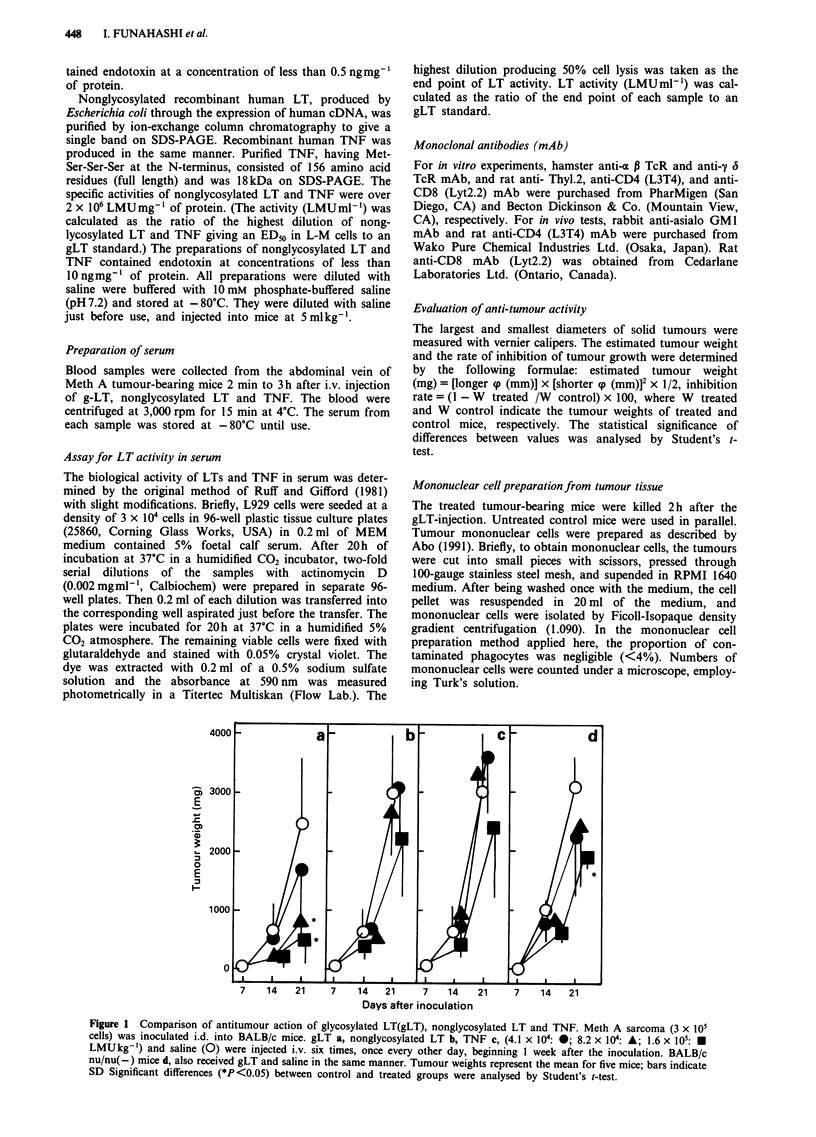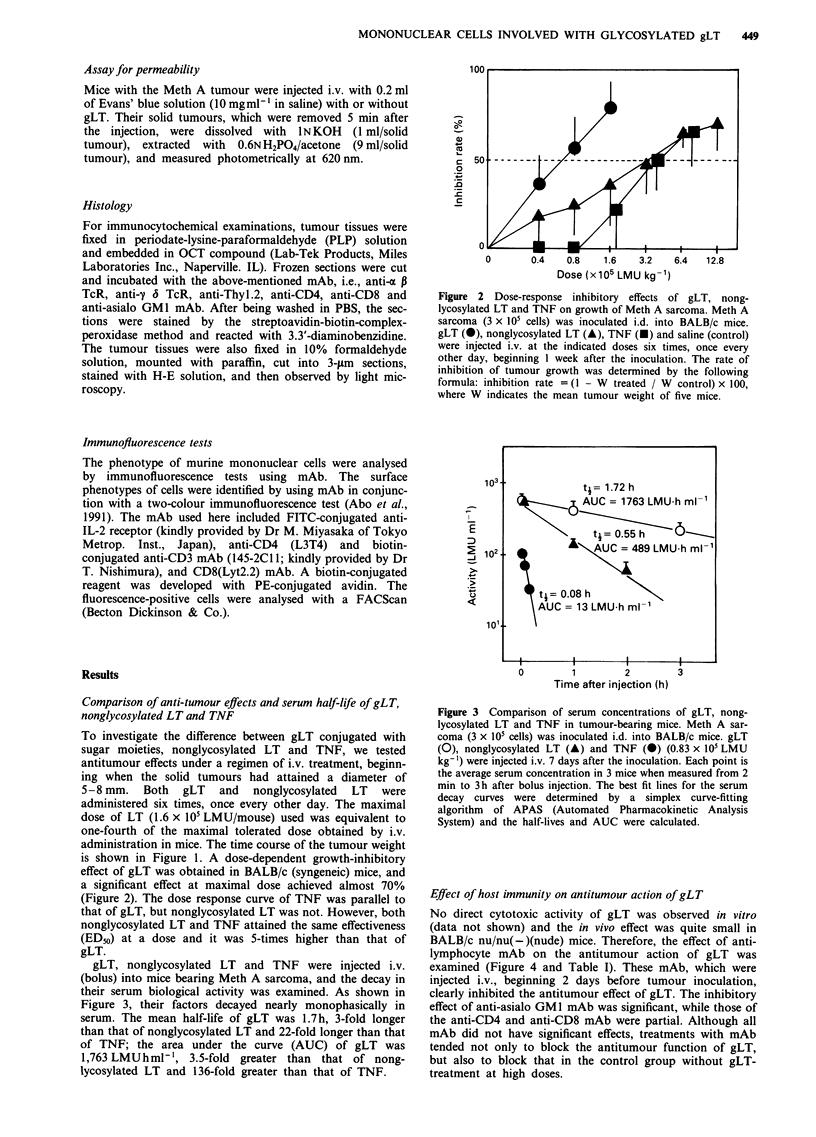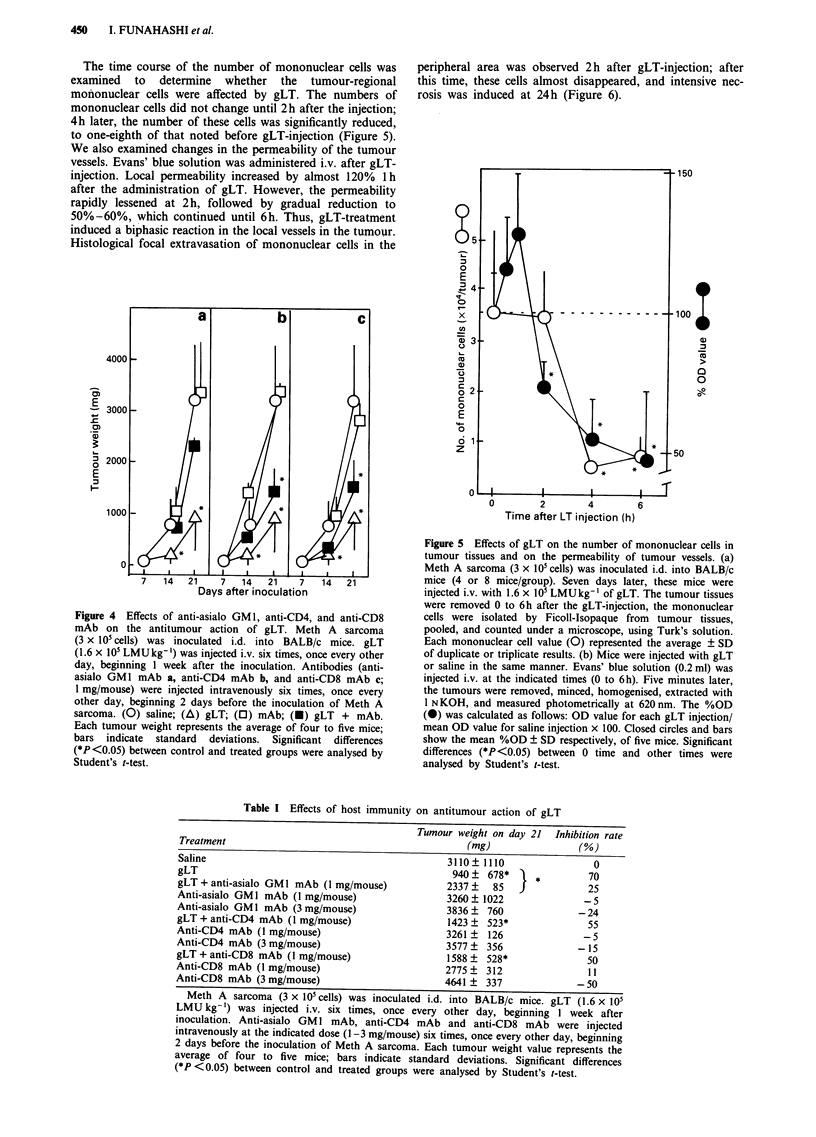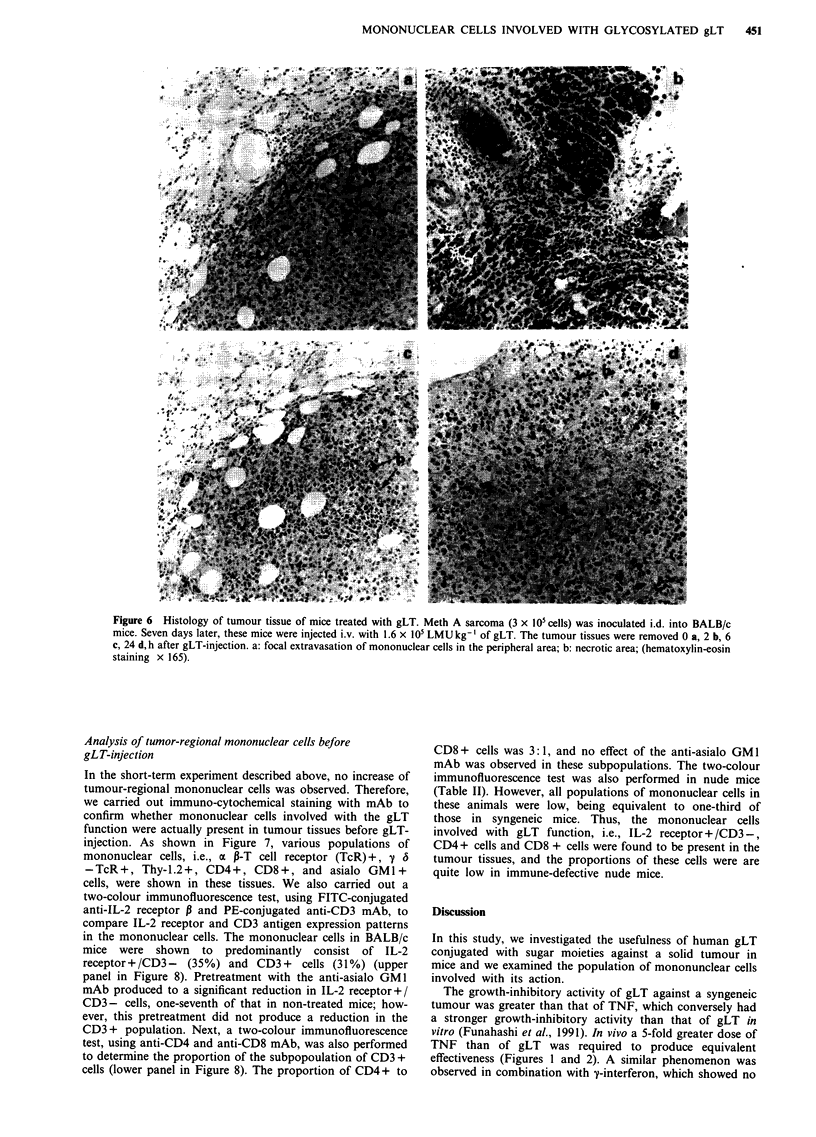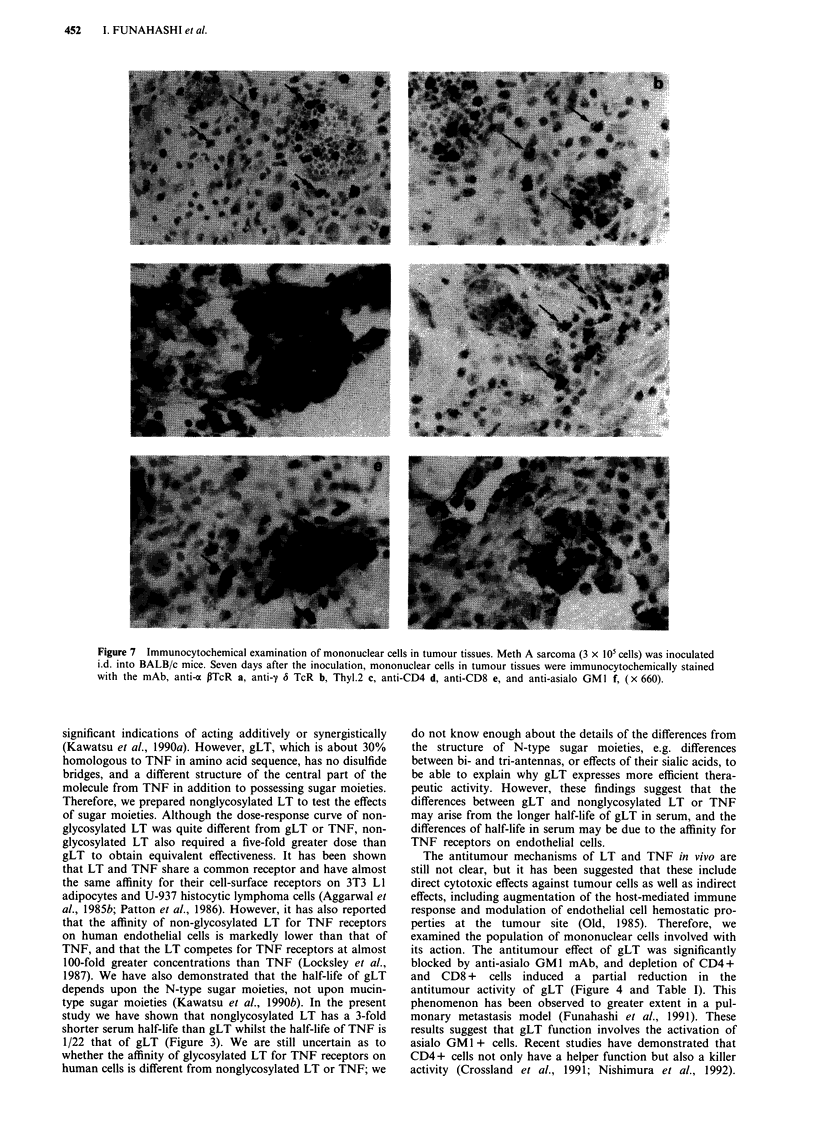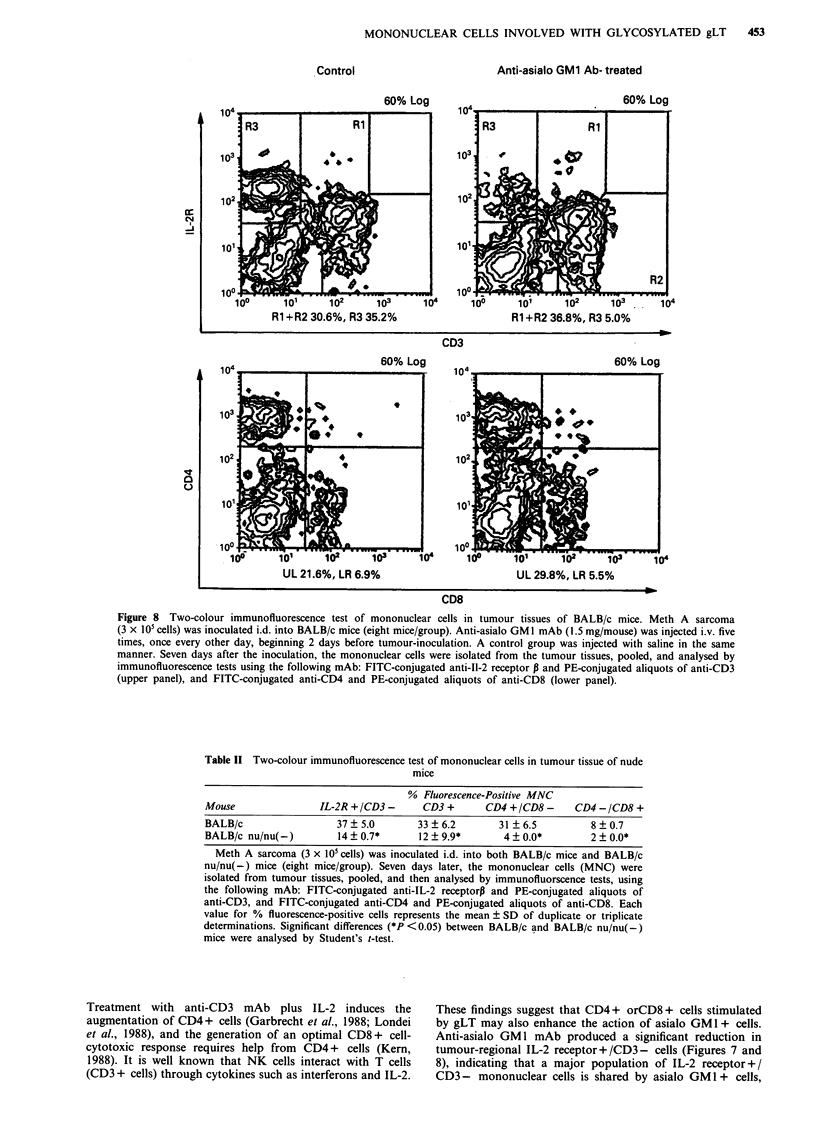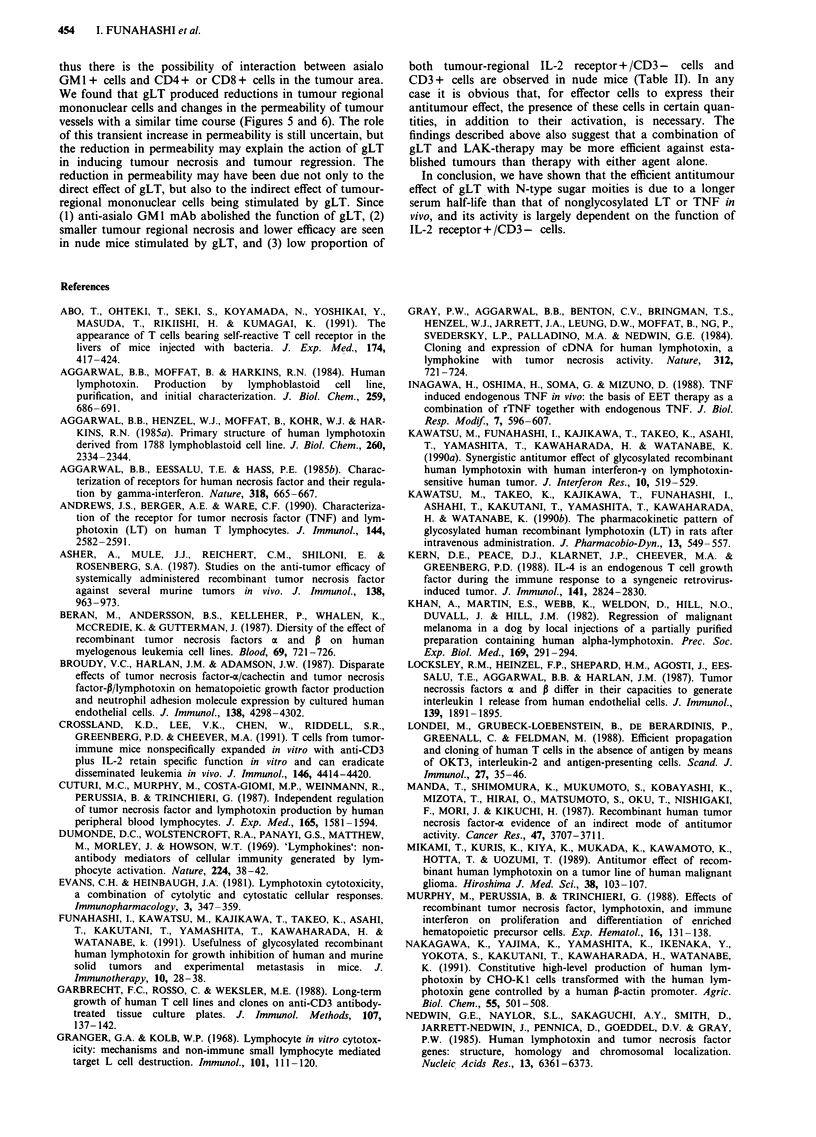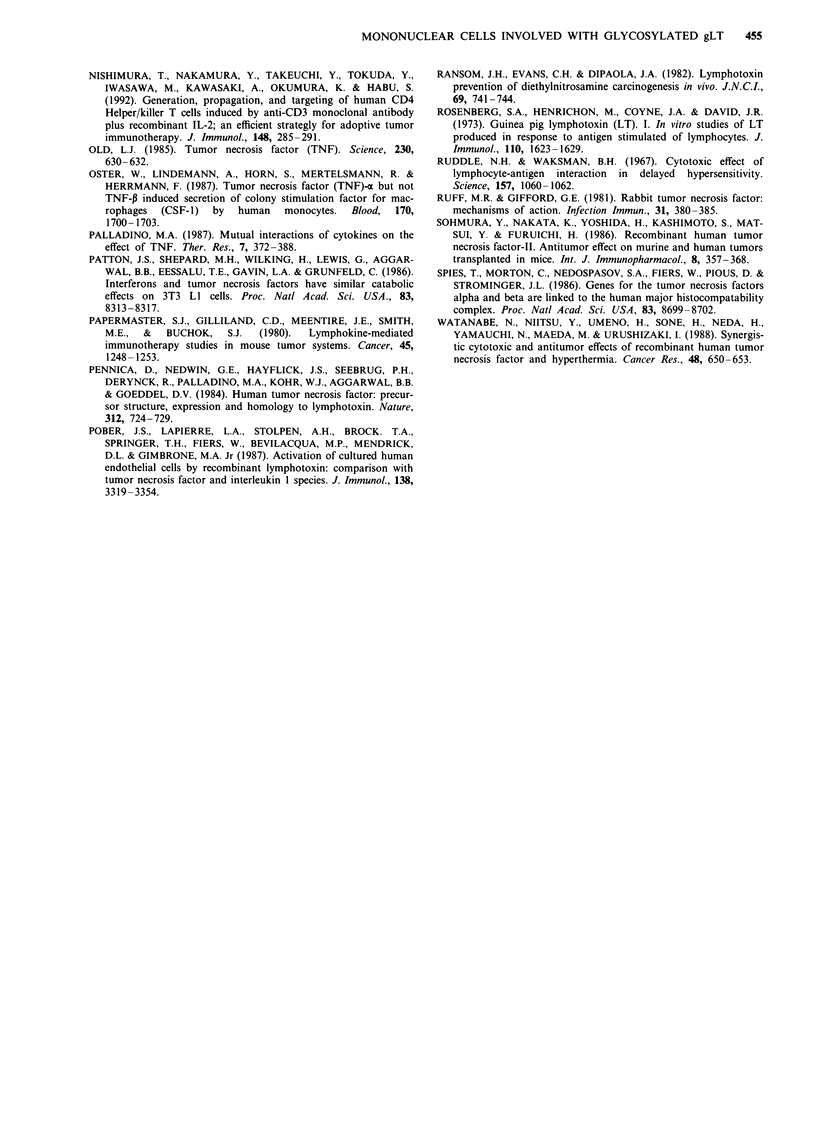# Tumour growth inhibition in mice by glycosylated recombinant human lymphotoxin: analysis of tumour-regional mononuclear cells involved with its action.

**DOI:** 10.1038/bjc.1993.86

**Published:** 1993-03

**Authors:** I. Funahashi, H. Watanabe, T. Abo, K. Indo, H. Miyaji

**Affiliations:** Department of Pathology, Hyogo College of Medicine, Japan.

## Abstract

**Images:**


Br. J. Cancer (1993), 67, 447–455 © Macmillan Press Ltd., 1993

### Tumour growth inhibition in mice by glycosylated recombinant human lymphotoxin: Analysis of tumour-regional mononuclear cells involved with its action

I. Funahashi[1], H. Watanabe[2], T. Abo[2], K. Indo[1] & H. Miyaji[1]

[1]*Department of Pathology, Hyogo College of Medicine,* [2]*Department of Immunology, Niigata University School of Medicine.*

**Summary** We compared the antitumour effects of glycosylated LT (gLT), nonglycosylated LT and TNF against a solid tumour in mice. We found that: (a) The systemic administration of gLT showed significant antitumour activity. These effects were, however, quite small in nude mice. Nonglycosylated LT and TNF attained the same degree of effectiveness as gLT, but at a 5-times higher dose. The serum half-life of gLT was 3-fold longer than that of nonglycosylated LT and 22-fold longer than that of TNF. (b) The effect of gLT was significantly blocked by pretreatment with anti-asialo GM1 antibody. Treatment with gLT produced a significant reduction in numbers of tumour-regional mononuclear cells, which in turn, produced increases intensive necrosis. (c) Mononuclear cells in the tumour tissues before gLT-injection were predominantly IL-2 receptor+/CD3− cells and CD3+ cells. Pretreatment with the anti-asialo GM1 antibody produced a drastic reduction of IL-2 receptor +/CD3 − cells. These findings suggest that the efficient antitumour effect of gLT is due to a longer serum half-life than that of nonglycosylated LT or TNF *in vivo*, and its function is largely mediated by IL-2 receptor+/CD3− cells.

Lymphotoxin (LT; TNFβ) was first characterised as a biological factor generated in response to T cell activation that could mediate the cytolysis of tumour cells and other targets (Ruddle & Wakoman, 1967; Granger & Kolb, 1968; Dumonde *et al.*, 1969; Rosenberg *et al.*, 1973; Evans & Heinbaugh, 1981). Later, human LT was purified and sequenced, and it was found to be about 30% homologous to tumour necrosis factor (TNF; TNFα) in its amino acid sequence (Aggarwal *et al.*, 1984, 1985*a*; Pennica *et al.*, 1984; Nedwin *et al.*, 1985). Furthermore, both LT and TNF were found to be linked to the human major histocompatibility complex (MHC) (Spies *et al.*, 1986), to share mainly a common cell-surface receptor (Aggarwal *et al.*, 1985*b*; Patton *et al.*, 1986), and to have similar biological activities (Gray *et al.*, 1984). However, LT and TNF differ in some aspects, e.g., in their affinity to TNF receptors, in their adhesive properties related to neutrophils and hematopoietic cells, in their expression of MHC class I antigen, and so on (Locksley *et al.*, 1987; Pober *et al.*, 1987; Broudy *et al.*, 1987; Andrews *et al.*, 1990; Murphy *et al.*, 1988; Oster *et al.*, 1987; Cuturi *et al.*, 1987; Beran *et al.*, 1987). It has also been reported that purified recombinant human TNF shows efficacy against tumours *in vivo* (Sohmura *et al.*, 1986; Palladino, 1987; Manda *et al.*, 1987; Asher *et al.*, 1987; Watanabe *et al.*, 1988; Inagawa *et al.*, 1988). However, there are few published data for *in vivo* studies of glycosylated human LT(gLT), (Khan *et al.*, 1982; Papermaster *et al.*, 1980; Ransom *et al.*, 1982), because the production of recombinant LT with sugar moieties was not achieved until quite recently. Human lymphotoxin genomic DNA has recently been cloned, and an efficient system for producing human gLT in Chinese hamster ovary cells has now been established in our laboratory (Nakagawa *et al.*, 1991). We have previously reported that gLT, unexpectedly, had weak cytotoxicity against tumour cells *in vitro*, but that it had a significant effect *in vivo* on solid tumours and metastases (Mikami *et al.*, 1989; Funahashi *et al.*, 1991). Combination therapy with human interferon γ can induce greater synergistic efficacy than combinations of TNF and interferon (Kawatsu *et al.*, 1990*a*). Our results suggest that host immunity is important for expressing the function of gLT. However, the detailed function of gLT has not been adequately explained. In this study, we demonstrated the effectiveness of human gLT against a solid tumour in mice, compared the results with those for nonglycosylated LT and TNF, and attempted to analyse tumour-regional mononuclear cells involved in its action.

### Materials and methods

### Mice

Female BALB/c and BALB/c nu/nu (−) mice, aged 6 weeks, were obtained from Japan SLC Inc. (Hamamatsu, Japan). They were fed a sterilised pellet diet and water *ad libitum* under pathogen-free conditions.

### Tumour

A Meth A fibrosarcoma was kindly provided by the Faculty of Pharmaceutical Sciences, Teikyo University; it was maintained in ascites form in syngeneic BALB/c mice by weekly intraperitoneal (i.p.) passage.

### LT and TNF

Recombinant human gLT, produced by Chinese hamster ovary cells through the expression of human LT genomic DNA, was purified and characterised as described previously (Nakagawa *et al.*, 1991). Purified LT, consisting mostly of 171 amino acid residues (full length), was composed of three species with molecular masses of 25, 23 (major) and 21 kDa (very minor) as judged by sodium dodecyl sulfate-polyacrylamide gel electrophoresis (SDS-PAGE). Amino acid and sugar components of these gLT species were analysed, and it was found that (1) 25 kDa LT has Leu-Pro-Gly-Val-Gly at N-terminus, carries both N-type sugar moieties (molecular ratio; Man:Fuc:Gal:GlucNAc:NANAc = 3:1.2:2.4: 4.0:1.3) and mucin-type sugar moieties (molecular ratio; GalNAc:Gal:NANAc = 1:1.06:1.14, and without Man), (2) 23 kDa carries N-type sugar moieties alone and comprises 80% of the preparation, and (3) 21 kDaLT is the same as the 23 kDa species except that it lacks 15 amino acid residues at the N-terminus, starts with Gln-His-Pro-Lys-Met sequence. The specific activity of the purified gLT preparation was over $2 \times 10^5$ LMU $mg^{-1}$ of protein in terms of cytotoxicity to L-M cells *in vitro*. (The concentration of gLT giving an $ED_{50}$ in L-M cells after culture was defined as 1 LMU $ml^{-1}$.) The gLT preparation used in this study con-

Correspondence: T. Kajikawa, Biochemical Research Laboratories, Kaneka Corporation 1-8, Miyamae, Takasago Hyogo 676, Japan.
Received 29 June 1992; and in revised form 11 September 1992.

tained endotoxin at a concentration of less than 0.5 $ng mg^{-1}$ of protein.

Nonglycosylated recombinant human LT, produced by *Escherichia coli* through the expression of human cDNA, was purified by ion-exchange column chromatography to give a single band on SDS-PAGE. Recombinant human TNF was produced in the same manner. Purified TNF, having Met-Ser-Ser-Ser at the N-terminus, consisted of 156 amino acid residues (full length) and was 18kDa on SDS-PAGE. The specific activities of nonglycosylated LT and TNF were over $2 \times 10^6$ LMU$mg^{-1}$ of protein. (The activity (LMU$ml^{-1}$) was calculated as the ratio of the highest dilution of nonglycosylated LT and TNF giving an $ED_{50}$ in L-M cells to an gLT standard.) The preparations of nonglycosylated LT and TNF contained endotoxin at concentrations of less than 10 $ng mg^{-1}$ of protein. All preparations were diluted with saline were buffered with 10 mM phosphate-buffered saline (pH7.2) and stored at −80°C. They were diluted with saline just before use, and injected into mice at 5 $ml kg^{-1}$.

*Preparation of serum*

Blood samples were collected from the abdominal vein of Meth A tumour-bearing mice 2 min to 3h after i.v. injection of g-LT, nonglycosylated LT and TNF. The blood were centrifuged at 3,000 rpm for 15 min at 4°C. The serum from each sample was stored at −80°C until use.

*Assay for LT activity in serum*

The biological activity of LTs and TNF in serum was determined by the original method of Ruff and Gifford (1981) with slight modifications. Briefly, L929 cells were seeded at a density of $3 \times 10^4$ cells in 96-well plastic tissue culture plates (25860, Corning Glass Works, USA) in 0.2 ml of MEM medium contained 5% foetal calf serum. After 20h of incubation at 37°C in a humidified $CO_2$ incubator, two-fold serial dilutions of the samples with actinomycin D (0.002 $mg ml^{-1}$, Calbiochem) were prepared in separate 96-well plates. Then 0.2 ml of each dilution was transferred into the corresponding well aspirated just before the transfer. The plates were incubated for 20h at 37°C in a humidified 5% $CO_2$ atmosphere. The remaining viable cells were fixed with glutaraldehyde and stained with 0.05% crystal violet. The dye was extracted with 0.2 ml of a 0.5% sodium sulfate solution and the absorbance at 590 nm was measured photometrically in a Titertec Multiskan (Flow Lab.). The highest dilution producing 50% cell lysis was taken as the end point of LT activity. LT activity (LMU$ml^{-1}$) was calculated as the ratio of the end point of each sample to an gLT standard.

*Monoclonal antibodies (mAb)*

For *in vitro* experiments, hamster anti-α β TcR and anti-γ δ TcR mAb, and rat anti- Thyl.2, anti-CD4 (L3T4), and anti-CD8 (Lyt2.2) mAb were purchased from PharMigen (San Diego, CA) and Becton Dickinson & Co. (Mountain View, CA), respectively. For *in vivo* tests, rabbit anti-asialo GM1 mAb and rat anti-CD4 (L3T4) mAb were purchased from Wako Pure Chemical Industries Ltd. (Osaka, Japan). Rat anti-CD8 mAb (Lyt2.2) was obtained from Cedarlane Laboratories Ltd. (Ontario, Canada).

*Evaluation of anti-tumour activity*

The largest and smallest diameters of solid tumours were measured with vernier calipers. The estimated tumour weight and the rate of inhibition of tumour growth were determined by the following formulae: estimated tumour weight (mg) = [longer φ (mm)] × [shorter φ (mm)]$^2$ × 1/2, inhibition rate = (1 − W treated /W control) × 100, where W treated and W control indicate the tumour weights of treated and control mice, respectively. The statistical significance of differences between values was analysed by Student's *t*-test.

*Mononuclear cell preparation from tumour tissue*

The treated tumour-bearing mice were killed 2h after the gLT-injection. Untreated control mice were used in parallel. Tumour mononuclear cells were prepared as described by Abo (1991). Briefly, to obtain mononuclear cells, the tumours were cut into small pieces with scissors, pressed through 100-gauge stainless steel mesh, and supended in RPMI 1640 medium. After being washed once with the medium, the cell pellet was resuspended in 20 ml of the medium, and mononuclear cells were isolated by Ficoll-Isopaque density gradient centrifugation (1.090). In the mononuclear cell preparation method applied here, the proportion of contaminated phagocytes was negligible (<4%). Numbers of mononuclear cells were counted under a microscope, employing Turk's solution.

**Figure 1** Comparison of antitumour action of glycosylated LT(gLT), nonglycosylated LT and TNF. Meth A sarcoma ($3 \times 10^5$ cells) was inoculated i.d. into BALB/c mice. gLT **a**, nonglycosylated LT **b**, TNF **c**, ($4.1 \times 10^4$: ●; $8.2 \times 10^4$: ▲; $1.6 \times 10^5$: ■ LMU$kg^{-1}$) and saline (○) were injected i.v. six times, once every other day, beginning 1 week after the inoculation. BALB/c nu/nu(−) mice **d**, also received gLT and saline in the same manner. Tumour weights represent the mean for five mice; bars indicate SD Significant differences (*$P$<0.05) between control and treated groups were analysed by Student's *t*-test.

*Assay for permeability*

Mice with the Meth A tumour were injected i.v. with 0.2 ml of Evans' blue solution (10 mg ml$^{-1}$ in saline) with or without gLT. Their solid tumours, which were removed 5 min after the injection, were dissolved with 1N KOH (1 ml/solid tumour), extracted with 0.6N $H_2PO_4$/acetone (9 ml/solid tumour), and measured photometrically at 620 nm.

*Histology*

For immunocytochemical examinations, tumour tissues were fixed in periodate-lysine-paraformaldehyde (PLP) solution and embedded in OCT compound (Lab-Tek Products, Miles Laboratories Inc., Naperville. IL). Frozen sections were cut and incubated with the above-mentioned mAb, i.e., anti-α β TcR, anti-γ δ TcR, anti-Thy1.2, anti-CD4, anti-CD8 and anti-asialo GM1 mAb. After being washed in PBS, the sections were stained by the streptoavidin-biotin-complex-peroxidase method and reacted with 3.3'-diaminobenzidine. The tumour tissues were also fixed in 10% formaldehyde solution, mounted with paraffin, cut into 3-μm sections, stained with H-E solution, and then observed by light microscopy.

*Immunofluorescence tests*

The phenotype of murine mononuclear cells were analysed by immunofluorescence tests using mAb. The surface phenotypes of cells were identified by using mAb in conjunction with a two-colour immunofluorescence test (Abo *et al.*, 1991). The mAb used here included FITC-conjugated anti-IL-2 receptor (kindly provided by Dr M. Miyasaka of Tokyo Metrop. Inst., Japan), anti-CD4 (L3T4) and biotin-conjugated anti-CD3 mAb (145-2C11; kindly provided by Dr T. Nishimura), and CD8(Lyt2.2) mAb. A biotin-conjugated reagent was developed with PE-conjugated avidin. The fluorescence-positive cells were analysed with a FACScan (Becton Dickinson & Co.).

### Results

*Comparison of anti-tumour effects and serum half-life of gLT, nonglycosylated LT and TNF*

To investigate the difference between gLT conjugated with sugar moieties, nonglycosylated LT and TNF, we tested antitumour effects under a regimen of i.v. treatment, beginning when the solid tumours had attained a diameter of 5–8 mm. Both gLT and nonglycosylated LT were administered six times, once every other day. The maximal dose of LT (1.6 × 10$^5$ LMU/mouse) used was equivalent to one-fourth of the maximal tolerated dose obtained by i.v. administration in mice. The time course of the tumour weight is shown in Figure 1. A dose-dependent growth-inhibitory effect of gLT was obtained in BALB/c (syngeneic) mice, and a significant effect at maximal dose achieved almost 70% (Figure 2). The dose response curve of TNF was parallel to that of gLT, but nonglycosylated LT was not. However, both nonglycosylated LT and TNF attained the same effectiveness ($ED_{50}$) at a dose and it was 5-times higher than that of gLT.

gLT, nonglycosylated LT and TNF were injected i.v. (bolus) into mice bearing Meth A sarcoma, and the decay in their serum biological activity was examined. As shown in Figure 3, their factors decayed nearly monophasically in serum. The mean half-life of gLT was 1.7h, 3-fold longer than that of nonglycosylated LT and 22-fold longer than that of TNF; the area under the curve (AUC) of gLT was 1,763 LMU h ml$^{-1}$, 3.5-fold greater than that of nonglycosylated LT and 136-fold greater than that of TNF.

**Figure 2** Dose-response inhibitory effects of gLT, nonglycosylated LT and TNF on growth of Meth A sarcoma. Meth A sarcoma (3 × 10$^5$ cells) was inoculated i.d. into BALB/c mice. gLT (●), nonglycosylated LT (▲), TNF (■) and saline (control) were injected i.v. at the indicated doses six times, once every other day, beginning 1 week after the inoculation. The rate of inhibition of tumour growth was determined by the following formula: inhibition rate = (1 – W treated / W control) × 100, where W indicates the mean tumour weight of five mice.

**Figure 3** Comparison of serum concentrations of gLT, nonglycosylated LT and TNF in tumour-bearing mice. Meth A sarcoma (3 × 10$^5$ cells) was inoculated i.d. into BALB/c mice. gLT (O), nonglycosylated LT (▲) and TNF (●) (0.83 × 10$^5$ LMU kg$^{-1}$) were injected i.v. 7 days after the inoculation. Each point is the average serum concentration in 3 mice when measured from 2 min to 3h after bolus injection. The best fit lines for the serum decay curves were determined by a simplex curve-fitting algorithm of APAS (Automated Pharmacokinetic Analysis System) and the half-lives and AUC were calculated.

*Effect of host immunity on antitumour action of gLT*

No direct cytotoxic activity of gLT was observed *in vitro* (data not shown) and the *in vivo* effect was quite small in BALB/c nu/nu(–)(nude) mice. Therefore, the effect of anti-lymphocyte mAb on the antitumour action of gLT was examined (Figure 4 and Table I). These mAb, which were injected i.v., beginning 2 days before tumour inoculation, clearly inhibited the antitumour effect of gLT. The inhibitory effect of anti-asialo GM1 mAb was significant, while those of the anti-CD4 and anti-CD8 mAb were partial. Although all mAb did not have significant effects, treatments with mAb tended not only to block the antitumour function of gLT, but also to block that in the control group without gLT-treatment at high doses.

The time course of the number of mononuclear cells was examined to determine whether the tumour-regional mononuclear cells were affected by gLT. The numbers of mononuclear cells did not change until 2h after the injection; 4h later, the number of these cells was significantly reduced, to one-eighth of that noted before gLT-injection (Figure 5). We also examined changes in the permeability of the tumour vessels. Evans' blue solution was administered i.v. after gLT-injection. Local permeability increased by almost 120% 1h after the administration of gLT. However, the permeability rapidly lessened at 2h, followed by gradual reduction to 50%–60%, which continued until 6h. Thus, gLT-treatment induced a biphasic reaction in the local vessels in the tumour. Histological focal extravasation of mononuclear cells in the peripheral area was observed 2h after gLT-injection; after this time, these cells almost disappeared, and intensive necrosis was induced at 24h (Figure 6).

**Figure 4** Effects of anti-asialo GM1, anti-CD4, and anti-CD8 mAb on the antitumour action of gLT. Meth A sarcoma ($3 \times 10^5$ cells) was inoculated i.d. into BALB/c mice. gLT ($1.6 \times 10^5$ LMU $kg^{-1}$) was injected i.v. six times, once every other day, beginning 1 week after the inoculation. Antibodies (anti-asialo GM1 mAb **a**, anti-CD4 mAb **b**, and anti-CD8 mAb **c**; 1 mg/mouse) were injected intravenously six times, once every other day, beginning 2 days before the inoculation of Meth A sarcoma. (○) saline; (△) gLT; (□) mAb; (■) gLT + mAb. Each tumour weight represents the average of four to five mice; bars indicate standard deviations. Significant differences (*$P<0.05$) between control and treated groups were analysed by Student's *t*-test.

**Figure 5** Effects of gLT on the number of mononuclear cells in tumour tissues and on the permeability of tumour vessels. (a) Meth A sarcoma ($3 \times 10^5$ cells) was inoculated i.d. into BALB/c mice (4 or 8 mice/group). Seven days later, these mice were injected i.v. with $1.6 \times 10^5$ LMU $kg^{-1}$ of gLT. The tumour tissues were removed 0 to 6h after the gLT-injection, the mononuclear cells were isolated by Ficoll-Isopaque from tumour tissues, pooled, and counted under a microscope, using Turk's solution. Each mononuclear cell value (○) represented the average ± SD of duplicate or triplicate results. (b) Mice were injected with gLT or saline in the same manner. Evans' blue solution (0.2 ml) was injected i.v. at the indicated times (0 to 6h). Five minutes later, the tumours were removed, minced, homogenised, extracted with 1 N KOH, and measured photometrically at 620 nm. The %OD (●) was calculated as follows: OD value for each gLT injection/mean OD value for saline injection × 100. Closed circles and bars show the mean %OD ± SD respectively, of five mice. Significant differences (*$P<0.05$) between 0 time and other times were analysed by Student's *t*-test.

**Table I** Effects of host immunity on antitumour action of gLT

| *Treatment* | *Tumour weight on day 21 (mg)* | *Inhibition rate (%)* |
|---|---|---|
| Saline | 3110 ± 1110 | 0 |
| gLT | 940 ± 678* } * | 70 |
| gLT + anti-asialo GM1 mAb (1 mg/mouse) | 2337 ± 85 } | 25 |
| Anti-asialo GM1 mAb (1 mg/mouse) | 3260 ± 1022 | −5 |
| Anti-asialo GM1 mAb (3 mg/mouse) | 3836 ± 760 | −24 |
| gLT + anti-CD4 mAb (1 mg/mouse) | 1423 ± 523* | 55 |
| Anti-CD4 mAb (1 mg/mouse) | 3261 ± 126 | −5 |
| Anti-CD4 mAb (3 mg/mouse) | 3577 ± 356 | −15 |
| gLT + anti-CD8 mAb (1 mg/mouse) | 1588 ± 528* | 50 |
| Anti-CD8 mAb (1 mg/mouse) | 2775 ± 312 | 11 |
| Anti-CD8 mAb (3 mg/mouse) | 4641 ± 337 | −50 |

Meth A sarcoma ($3 \times 10^5$ cells) was inoculated i.d. into BALB/c mice. gLT ($1.6 \times 10^5$ LMU $kg^{-1}$) was injected i.v. six times, once every other day, beginning 1 week after inoculation. Anti-asialo GM1 mAb, anti-CD4 mAb and anti-CD8 mAb were injected intravenously at the indicated dose (1–3 mg/mouse) six times, once every other day, beginning 2 days before the inoculation of Meth A sarcoma. Each tumour weight value represents the average of four to five mice; bars indicate standard deviations. Significant differences (*$P<0.05$) between control and treated groups were analysed by Student's *t*-test.

**Figure 6** Histology of tumour tissue of mice treated with gLT. Meth A sarcoma ($3 \times 10^5$ cells) was inoculated i.d. into BALB/c mice. Seven days later, these mice were injected i.v. with $1.6 \times 10^5$ LMU kg$^{-1}$ of gLT. The tumour tissues were removed 0 **a**, 2 **b**, 6 **c**, 24 **d**, h after gLT-injection. a: focal extravasation of mononuclear cells in the peripheral area; b: necrotic area; (hematoxylin-eosin staining $\times$ 165).

*Analysis of tumor-regional mononuclear cells before gLT-injection*

In the short-term experiment described above, no increase of tumour-regional mononuclear cells was observed. Therefore, we carried out immuno-cytochemical staining with mAb to confirm whether mononuclear cells involved with the gLT function were actually present in tumour tissues before gLT-injection. As shown in Figure 7, various populations of mononuclear cells, i.e., $\alpha$ $\beta$-T cell receptor (TcR)+, $\gamma$ $\delta$ −TcR+, Thy-1.2+, CD4+, CD8+, and asialo GM1+ cells, were shown in these tissues. We also carried out a two-colour immunofluorescence test, using FITC-conjugated anti-IL-2 receptor $\beta$ and PE-conjugated anti-CD3 mAb, to compare IL-2 receptor and CD3 antigen expression patterns in the mononuclear cells. The mononuclear cells in BALB/c mice were shown to predominantly consist of IL-2 receptor+/CD3− (35%) and CD3+ cells (31%) (upper panel in Figure 8). Pretreatment with the anti-asialo GM1 mAb produced to a significant reduction in IL-2 receptor+/CD3− cells, one-seventh of that in non-treated mice; however, this pretreatment did not produce a reduction in the CD3+ population. Next, a two-colour immunofluorescence test, using anti-CD4 and anti-CD8 mAb, was also performed to determine the proportion of the subpopoulation of CD3+ cells (lower panel in Figure 8). The proportion of CD4+ to CD8+ cells was 3:1, and no effect of the anti-asialo GM1 mAb was observed in these subpopulations. The two-colour immunofluorescence test was also performed in nude mice (Table II). However, all populations of mononuclear cells in these animals were low, being equivalent to one-third of those in syngeneic mice. Thus, the mononuclear cells involved with gLT function, i.e., IL-2 receptor+/CD3−, CD4+ cells and CD8 + cells were found to be present in the tumour tissues, and the proportions of these cells were are quite low in immune-defective nude mice.

### Discussion

In this study, we investigated the usefulness of human gLT conjugated with sugar moieties against a solid tumour in mice and we examined the population of mononunclear cells involved with its action.

The growth-inhibitory activity of gLT against a syngeneic tumour was greater than that of TNF, which conversely had a stronger growth-inhibitory activity than that of gLT *in vitro* (Funahashi *et al.*, 1991). *In vivo* a 5-fold greater dose of TNF than of gLT was required to produce equivalent effectiveness (Figures 1 and 2). A similar phenomenon was observed in combination with $\gamma$-interferon, which showed no

**Figure 7** Immunocytochemical examination of mononuclear cells in tumour tissues. Meth A sarcoma ($3 \times 10^5$ cells) was inoculated i.d. into BALB/c mice. Seven days after the inoculation, mononuclear cells in tumour tissues were immunocytochemically stained with the mAb, anti-α βTcR **a**, anti-γ δ TcR **b**, Thyl.2 **c**, anti-CD4 **d**, anti-CD8 **e**, and anti-asialo GM1 **f**, (× 660).

significant indications of acting additively or synergistically (Kawatsu *et al.*, 1990*a*). However, gLT, which is about 30% homologous to TNF in amino acid sequence, has no disulfide bridges, and a different structure of the central part of the molecule from TNF in addition to possessing sugar moieties. Therefore, we prepared nonglycosylated LT to test the effects of sugar moieties. Although the dose-response curve of nonglycosylated LT was quite different from gLT or TNF, nonglycosylated LT also required a five-fold greater dose than gLT to obtain equivalent effectiveness. It has been shown that LT and TNF share a common receptor and have almost the same affinity for their cell-surface receptors on 3T3 L1 adipocytes and U-937 histocytic lymphoma cells (Aggarwal *et al.*, 1985*b*; Patton *et al.*, 1986). However, it has also reported that the affinity of non-glycosylated LT for TNF receptors on human endothelial cells is markedly lower than that of TNF, and that the LT competes for TNF receptors at almost 100-fold greater concentrations than TNF (Locksley *et al.*, 1987). We have also demonstrated that the half-life of gLT depends upon the N-type sugar moieties, not upon mucin-type sugar moieties (Kawatsu *et al.*, 1990*b*). In the present study we have shown that nonglycosylated LT has a 3-fold shorter serum half-life than gLT whilst the half-life of TNF is 1/22 that of gLT (Figure 3). We are still uncertain as to whether the affinity of glycosylated LT for TNF receptors on human cells is different from nonglycosylated LT or TNF; we do not know enough about the details of the differences from the structure of N-type sugar moieties, e.g. differences between bi- and tri-antennas, or effects of their sialic acids, to be able to explain why gLT expresses more efficient therapeutic activity. However, these findings suggest that the differences between gLT and nonglycosylated LT or TNF may arise from the longer half-life of gLT in serum, and the differences of half-life in serum may be due to the affinity for TNF receptors on endothelial cells.

The antitumour mechanisms of LT and TNF *in vivo* are still not clear, but it has been suggested that these include direct cytotoxic effects against tumour cells as well as indirect effects, including augmentation of the host-mediated immune response and modulation of endothelial cell hemostatic properties at the tumour site (Old, 1985). Therefore, we examined the population of mononuclear cells involved with its action. The antitumour effect of gLT was significantly blocked by anti-asialo GM1 mAb, and depletion of CD4+ and CD8+ cells induced a partial reduction in the antitumour activity of gLT (Figure 4 and Table I). This phenomenon has been observed to greater extent in a pulmonary metastasis model (Funahashi *et al.*, 1991). These results suggest that gLT function involves the activation of asialo GM1+ cells. Recent studies have demonstrated that CD4+ cells not only have a helper function but also a killer activity (Crossland *et al.*, 1991; Nishimura *et al.*, 1992).

**Figure 8** Two-colour immunofluorescence test of mononuclear cells in tumour tissues of BALB/c mice. Meth A sarcoma ($3 \times 10^5$ cells) was inoculated i.d. into BALB/c mice (eight mice/group). Anti-asialo GM1 mAb (1.5 mg/mouse) was injected i.v. five times, once every other day, beginning 2 days before tumour-inoculation. A control group was injected with saline in the same manner. Seven days after the inoculation, the mononuclear cells were isolated from the tumour tissues, pooled, and analysed by immunofluorescence tests using the following mAb: FITC-conjugated anti-Il-2 receptor $\beta$ and PE-conjugated aliquots of anti-CD3 (upper panel), and FITC-conjugated anti-CD4 and PE-conjugated aliquots of anti-CD8 (lower panel).

**Table II** Two-colour immunofluorescence test of mononuclear cells in tumour tissue of nude mice

| | *% Fluorescence-Positive MNC* | | | |
|---|---|---|---|---|
| *Mouse* | *IL-2R + /CD3 −* | *CD3 +* | *CD4 + /CD8 −* | *CD4 − /CD8 +* |
| BALB/c | 37 ± 5.0 | 33 ± 6.2 | 31 ± 6.5 | 8 ± 0.7 |
| BALB/c nu/nu(−) | 14 ± 0.7* | 12 ± 9.9* | 4 ± 0.0* | 2 ± 0.0* |

Meth A sarcoma ($3 \times 10^5$ cells) was inoculated i.d. into both BALB/c mice and BALB/c nu/nu(−) mice (eight mice/group). Seven days later, the mononuclear cells (MNC) were isolated from tumour tissues, pooled, and then analysed by immunofluorscence tests, using the following mAb: FITC-conjugated anti-IL-2 receptor$\beta$ and PE-conjugated aliquots of anti-CD3, and FITC-conjugated anti-CD4 and PE-conjugated aliquots of anti-CD8. Each value for % fluorescence-positive cells represents the mean ± SD of duplicate or triplicate determinations. Significant differences ($*P < 0.05$) between BALB/c and BALB/c nu/nu(−) mice were analysed by Student's *t*-test.

Treatment with anti-CD3 mAb plus IL-2 induces the augmentation of CD4+ cells (Garbrecht *et al.*, 1988; Londei *et al.*, 1988), and the generation of an optimal CD8+ cell-cytotoxic response requires help from CD4+ cells (Kern, 1988). It is well known that NK cells interact with T cells (CD3+ cells) through cytokines such as interferons and IL-2. These findings suggest that CD4+ orCD8+ cells stimulated by gLT may also enhance the action of asialo GM1+ cells. Anti-asialo GM1 mAb produced a significant reduction in tumour-regional IL-2 receptor+/CD3− cells (Figures 7 and 8), indicating that a major population of IL-2 receptor+/CD3− mononuclear cells is shared by asialo GM1+ cells, thus there is the possibility of interaction between asialo GM1+ cells and CD4+ or CD8+ cells in the tumour area. We found that gLT produced reductions in tumour regional mononuclear cells and changes in the permeability of tumour vessels with a similar time course (Figures 5 and 6). The role of this transient increase in permeability is still uncertain, but the reduction in permeability may explain the action of gLT in inducing tumour necrosis and tumour regression. The reduction in permeability may have been due not only to the direct effect of gLT, but also to the indirect effect of tumour-regional mononuclear cells being stimulated by gLT. Since (1) anti-asialo GM1 mAb abolished the function of gLT, (2) smaller tumour regional necrosis and lower efficacy are seen in nude mice stimulated by gLT, and (3) low proportion of both tumour-regional IL-2 receptor+/CD3− cells and CD3+ cells are observed in nude mice (Table II). In any case it is obvious that, for effector cells to express their antitumour effect, the presence of these cells in certain quantities, in addition to their activation, is necessary. The findings described above also suggest that a combination of gLT and LAK-therapy may be more efficient against established tumours than therapy with either agent alone.

In conclusion, we have shown that the efficient antitumour effect of gLT with N-type sugar moities is due to a longer serum half-life than that of nonglycosylated LT or TNF *in vivo*, and its activity is largely dependent on the function of IL-2 receptor+/CD3− cells.

### References

ABO, T., OHTEKI, T., SEKI, S., KOYAMADA, N., YOSHIKAI, Y., MASUDA, T., RIKIISHI, H. & KUMAGAI, K. (1991). The appearance of T cells bearing self-reactive T cell receptor in the livers of mice injected with bacteria. *J. Exp. Med.*, **174**, 417–424.

AGGARWAL, B.B., MOFFAT, B. & HARKINS, R.N. (1984). Human lymphotoxin. Production by lymphoblastoid cell line, purification, and initial characterization. *J. Biol. Chem.*, **259**, 686–691.

AGGARWAL, B.B., HENZEL, W.J., MOFFAT, B., KOHR, W.J. & HARKINS, R.N. (1985*a*). Primary structure of human lymphotoxin derived from 1788 lymphoblastoid cell line. *J. Biol. Chem.*, **260**, 2334–2344.

AGGARWAL, B.B., EESSALU, T.E. & HASS, P.E. (1985*b*). Characterization of receptors for human necrosis factor and their regulation by gamma-interferon. *Nature*, **318**, 665–667.

ANDREWS, J.S., BERGER, A.E. & WARE, C.F. (1990). Characterization of the receptor for tumor necrosis factor (TNF) and lymphotoxin (LT) on human T lymphocytes. *J. Immunol.*, **144**, 2582–2591.

ASHER, A., MULE, J.J., REICHERT, C.M., SHILONI, E. & ROSENBERG, S.A. (1987). Studies on the anti-tumor efficacy of systemically administered recombinant tumor necrosis factor against several murine tumors *in vivo*. *J. Immunol.*, **138**, 963–973.

BERAN, M., ANDERSSON, B.S., KELLEHER, P., WHALEN, K., MCCREDIE, K. & GUTTERMAN, J. (1987). Diersity of the effect of recombinant tumor necrosis factors α and β on human myelogenous leukemia cell lines. *Blood*, **69**, 721–726.

BROUDY, V.C., HARLAN, J.M. & ADAMSON, J.W. (1987). Disparate effects of tumor necrosis factor-α/cachectin and tumor necrosis factor-β/lymphotoxin on hematopoietic growth factor production and neutrophil adhesion molecule expression by cultured human endothelial cells. *J. Immunol.*, **138**, 4298–4302.

CROSSLAND, K.D., LEE, V.K., CHEN, W., RIDDELL, S.R., GREENBERG, P.D. & CHEEVER, M.A. (1991). T cells from tumor-immune mice nonspecifically expanded *in vitro* with anti-CD3 plus IL-2 retain specific function *in vitro* and can eradicate disseminated leukemia *in vivo*. *J. Immunol.*, **146**, 4414–4420.

CUTURI, M.C., MURPHY, M., COSTA-GIOMI, M.P., WEINMANN, R., PERUSSIA, B. & TRINCHIERI, G. (1987). Independent regulation of tumor necrosis factor and lymphotoxin production by human peripheral blood lymphocytes. *J. Exp. Med.*, **165**, 1581–1594.

DUMONDE, D.C., WOLSTENCROFT, R.A., PANAYI, G.S., MATTHEW, M., MORLEY, J. & HOWSON, W.T. (1969). 'Lymphokines': non-antibody mediators of cellular immunity generated by lymphocyte activation. *Nature*, **224**, 38–42.

EVANS, C.H. & HEINBAUGH, J.A. (1981). Lymphotoxin cytotoxicity, a combination of cytolytic and cytostatic cellular responses. *Immunopharmacology*, **3**, 347–359.

FUNAHASHI, I., KAWATSU, M., KAJIKAWA, T., TAKEO, K., ASAHI, T., KAKUTANI, T., YAMASHITA, T., KAWAHARADA, H. & WATANABE, k. (1991). Usefulness of glycosylated recombinant human lymphotoxin for growth inhibition of human and murine solid tumors and experimental metastasis in mice. *J. Immunotherapy*, **10**, 28–38.

GARBRECHT, F.C., ROSSO, C. & WEKSLER, M.E. (1988). Long-term growth of human T cell lines and clones on anti-CD3 antibody-treated tissue culture plates. *J. Immunol. Methods*, **107**, 137–142.

GRANGER, G.A. & KOLB, W.P. (1968). Lymphocyte *in vitro* cytotoxicity: mechanisms and non-immune small lymphocyte mediated target L cell destruction. *Immunol.*, **101**, 111–120.

GRAY, P.W., AGGARWAL, B.B., BENTON, C.V., BRINGMAN, T.S., HENZEL, W.J., JARRETT, J.A., LEUNG, D.W., MOFFAT, B., NG, P., SVEDERSKY, L.P., PALLADINO, M.A. & NEDWIN, G.E. (1984). Cloning and expression of cDNA for human lymphotoxin, a lymphokine with tumor necrosis activity. *Nature*, **312**, 721–724.

INAGAWA, H., OSHIMA, H., SOMA, G. & MIZUNO, D. (1988). TNF induced endogenous TNF *in vivo*: the basis of EET therapy as a combination of rTNF together with endogenous TNF. *J. Biol. Resp. Modif.*, **7**, 596–607.

KAWATSU, M., FUNAHASHI, I., KAJIKAWA, T., TAKEO, K., ASAHI, T., YAMASHITA, T., KAWAHARADA, H. & WATANABE, K. (1990*a*). Synergistic antitumor effect of glycosylated recombinant human lymphotoxin with human interferon-γ on lymphotoxin-sensitive human tumor. *J. Interferon Res.*, **10**, 519–529.

KAWATSU, M., TAKEO, K., KAJIKAWA, T., FUNAHASHI, I., ASHAHI, T., KAKUTANI, T., YAMASHITA, T., KAWAHARADA, H. & WATANABE, K. (1990*b*). The pharmacokinetic pattern of glycosylated human recombinant lymphotoxin (LT) in rats after intravenous administration. *J. Pharmacobio-Dyn.*, **13**, 549–557.

KERN, D.E., PEACE, D.J., KLARNET, J.P., CHEEVER, M.A. & GREENBERG, P.D. (1988). IL-4 is an endogenous T cell growth factor during the immune response to a syngeneic retrovirus-induced tumor. *J. Immunol.*, **141**, 2824–2830.

KHAN, A., MARTIN, E.S., WEBB, K., WELDON, D., HILL, N.O., DUVALL, J. & HILL, J.M. (1982). Regression of malignant melanoma in a dog by local injections of a partially purified preparation containing human alpha-lymphotoxin. *Prec. Soc. Exp. Biol. Med.*, **169**, 291–294.

LOCKSLEY, R.M., HEINZEL, F.P., SHEPARD, H.M., AGOSTI, J., EESSALU, T.E., AGGARWAL, B.B. & HARLAN, J.M. (1987). Tumor necrossis factors α and β differ in their capacities to generate interleukin 1 release from human endothelial cells. *J. Immunol.*, **139**, 1891–1895.

LONDEI, M., GRUBECK-LOEBENSTEIN, B., DE BERARDINIS, P., GREENALL, C. & FELDMAN, M. (1988). Efficient propagation and cloning of human T cells in the absence of antigen by means of OKT3, interleukin-2 and antigen-presenting cells. *Scand. J. Immunol.*, **27**, 35–46.

MANDA, T., SHIMOMURA, K., MUKUMOTO, S., KOBAYASHI, K., MIZOTA, T., HIRAI, O., MATSUMOTO, S., OKU, T., NISHIGAKI, F., MORI, J. & KIKUCHI, H. (1987). Recombinant human tumor necrosis factor-α evidence of an indirect mode of antitumor activity. *Cancer Res.*, **47**, 3707–3711.

MIKAMI, T., KURIS, K., KIYA, K., MUKADA, K., KAWAMOTO, K., HOTTA, T. & UOZUMI, T. (1989). Antitumor effect of recombinant human lymphotoxin on a tumor line of human malignant glioma. *Hiroshima J. Med. Sci.*, **38**, 103–107.

MURPHY, M., PERUSSIA, B. & TRINCHIERI, G. (1988). Effects of recombinant tumor necrosis factor, lymphotoxin, and immune interferon on proliferation and differentiation of enriched hematopoietic precursor cells. *Exp. Hematol.*, **16**, 131–138.

NAKAGAWA, K., YAJIMA, K., YAMASHITA, K., IKENAKA, Y., YOKOTA, S., KAKUTANI, T., KAWAHARADA, H., WATANABE, K. (1991). Constitutive high-level production of human lymphotoxin by CHO-K1 cells transformed with the human lymphotoxin gene controlled by a human β-actin promoter. *Agric. Biol. Chem.*, **55**, 501–508.

NEDWIN, G.E., NAYLOR, S.L., SAKAGUCHI, A.Y., SMITH, D., JARRETT-NEDWIN, J., PENNICA, D., GOEDDEL, D.V. & GRAY, P.W. (1985). Human lymphotoxin and tumor necrosis factor genes: structure, homology and chromosomal localization. *Nucleic Acids Res.*, **13**, 6361–6373.

NISHIMURA, T., NAKAMURA, Y., TAKEUCHI, Y., TOKUDA, Y., IWASAWA, M., KAWASAKI, A., OKUMURA, K. & HABU, S. (1992). Generation, propagation, and targeting of human CD4 Helper/killer T cells induced by anti-CD3 monoclonal antibody plus recombinant IL-2; an efficient strategly for adoptive tumor immunotherapy. *J. Immunol.*, **148**, 285-291.

OLD, L.J. (1985). Tumor necrosis factor (TNF). *Science*, **230**, 630-632.

OSTER, W., LINDEMANN, A., HORN, S., MERTELSMANN, R. & HERRMANN, F. (1987). Tumor necrosis factor (TNF)-α but not TNF-β induced secretion of colony stimulation factor for macrophages (CSF-1) by human monocytes. *Blood*, **170**, 1700-1703.

PALLADINO, M.A. (1987). Mutual interactions of cytokines on the effect of TNF. *Ther. Res.*, **7**, 372-388.

PATTON, J.S., SHEPARD, M.H., WILKING, H., LEWIS, G., AGGARWAL, B.B., EESSALU, T.E., GAVIN, L.A. & GRUNFELD, C. (1986). Interferons and tumor necrosis factors have similar catabolic effects on 3T3 L1 cells. *Proc. Natl Acad. Sci. USA.*, **83**, 8313-8317.

PAPERMASTER, S.J., GILLILAND, C.D., MEENTIRE, J.E., SMITH, M.E., & BUCHOK, S.J. (1980). Lymphokine-mediated immunotherapy studies in mouse tumor systems. *Cancer*, **45**, 1248-1253.

PENNICA, D., NEDWIN, G.E., HAYFLICK, J.S., SEEBRUG, P.H., DERYNCK, R., PALLADINO, M.A., KOHR, W.J., AGGARWAL, B.B. & GOEDDEL, D.V. (1984). Human tumor necrosis factor: precursor structure, expression and homology to lymphotoxin. *Nature*, **312**, 724-729.

POBER, J.S., LAPIERRE, L.A., STOLPEN, A.H., BROCK. T.A., SPRINGER, T.H., FIERS, W., BEVILACQUA, M.P., MENDRICK, D.L. & GIMBRONE, M.A. Jr (1987). Activation of cultured human endothelial cells by recombinant lymphotoxin: comparison with tumor necrosis factor and interleukin 1 species. *J. Immunol.*, **138**, 3319-3354.

RANSOM, J.H., EVANS, C.H. & DIPAOLA, J.A. (1982). Lymphotoxin prevention of diethylnitrosamine carcinogenesis *in vivo*. *J.N.C.I.*, **69**, 741-744.

ROSENBERG, S.A., HENRICHON, M., COYNE, J.A. & DAVID, J.R. (1973). Guinea pig lymphotoxin (LT). I. *In vitro* studies of LT produced in response to antigen stimulated of lymphocytes. *J. Immunol.*, **110**, 1623-1629.

RUDDLE, N.H. & WAKSMAN, B.H. (1967). Cytotoxic effect of lymphocyte-antigen interaction in delayed hypersensitivity. *Science*, **157**, 1060-1062.

RUFF, M.R. & GIFFORD, G.E. (1981). Rabbit tumor necrosis factor: mechanisms of action. *Infection Immun.*, **31**, 380-385.

SOHMURA, Y., NAKATA, K., YOSHIDA, H., KASHIMOTO, S., MATSUI, Y. & FURUICHI, H. (1986). Recombinant human tumor necrosis factor-II. Antitumor effect on murine and human tumors transplanted in mice. *Int. J. Immunopharmacol.*, **8**, 357-368.

SPIES, T., MORTON, C., NEDOSPASOV, S.A., FIERS, W., PIOUS, D. & STROMINGER, J.L. (1986). Genes for the tumor necrosis factors alpha and beta are linked to the human major histocompatability complex. *Proc. Natl Acad. Sci. USA*, **83**, 8699-8702.

WATANABE, N., NIITSU, Y., UMENO, H., SONE, H., NEDA, H., YAMAUCHI, N., MAEDA, M. & URUSHIZAKI, I. (1988). Synergistic cytotoxic and antitumor effects of recombinant human tumor necrosis factor and hyperthermia. *Cancer Res.*, **48**, 650-653.